# Supplementary material for: Assessment of compliance with quality laboratory standards in medical laboratories within the Kumasi Metropolis, Ghana
Source: PLoS One. 2026 Mar 20;21(3):e0335380. doi: 10.1371/journal.pone.0335380 (PMC13004333; doi:10.1371/journal.pone.0335380)
Supplement: Attachment — Submitted filename: Review Comments.docx [file pone.0335380.s002.docx]

**Review Comments**

**Manuscript ID:** PONE-D-25-53279
**Title:** Assessment of compliance with quality laboratory standards in medical laboratories within the Kumasi Metropolis, Ghana
**Journal:** PLOS ONE

**General Assessment**

The manuscript evaluates compliance with quality laboratory standards among medical laboratories in the Kumasi Metropolis using the WHO SLIPTA checklist. This is a highly relevant topic for laboratory system strengthening in low- and middle-income countries and aligns well with the scope of PLOS ONE.

Key strengths include the use of a recognized international assessment framework and the attempt to assess all eligible laboratories within the study area. Nonetheless, important aspects of the **Methods**, **Results**, and **Discussion** sections require clearer explanation to ensure that readers can fully understand how scores were generated, how facilities were selected, and how conclusions were derived from the data.

**Major Comments**

**1. SLIPTA Scoring, Categories, and Interpretation**

The manuscript reports overall and domain-specific compliance scores (e.g., a median score of 61) without clearly explaining how these relate to the maximum SLIPTA score of 275 or to the official SLIPTA star-rating system. For readers unfamiliar with SLIPTA, the practical meaning of these values is difficult to interpret.

In addition, the manuscript introduces custom compliance categories (low, moderate, high) that do not directly correspond to the standard 0–5 star SLIPTA bands, and the rationale for these cut-offs is not clearly justified.

**Recommendations:**

- Explicitly state whether analyses were conducted using raw scores, percentages of the maximum score, or star equivalents.
- Clearly link observed score ranges to the official SLIPTA star levels.
- Consider presenting a table or figure showing the distribution of laboratories across SLIPTA star categories.
- If non-standard low/moderate/high categories are retained, provide a clear justification for the chosen thresholds and apply them consistently throughout the Results and Discussion.

**2. Significance, Originality, and Contribution to the Literature**

The manuscript documents low levels of compliance with laboratory quality standards in a large metropolitan area and highlights staffing and phlebotomy gaps. However, similar SLIPTA-based assessments have been conducted in Ghana and other African settings, and the added value of this study is not sufficiently articulated.

**Recommendations:**

- Clearly state in the Introduction what is new about this study (e.g., census coverage, detailed analysis of quality system essentials, explicit linkage to staffing norms).
- Avoid overstating uniqueness; instead, emphasize the contextual and operational contributions to Ghana’s laboratory network and policy implementation.

**3. Study Setting, Sampling Frame, and External Validity**

While a census approach is reported, the total number of eligible laboratories and the source of the sampling frame are not clearly described. There is also inconsistency between the stated inclusion of hospital laboratories and the reporting of private standalone laboratories in the Results.

Additionally, only one tertiary facility was included, while private facilities dominate the sample.

**Recommendations:**

- Clearly describe how the sampling frame was constructed (e.g., Ghana Health Service lists, regulatory registers, professional councils).
- State the total number of eligible laboratories by type and ownership and indicate whether any facilities were excluded or declined participation.
- Expand the limitations section to discuss how the facility mix and limited number of tertiary laboratories affect generalizability.

**4. Measurement Tool (SLIPTA)**

The introduction of non-standard compliance categories (low, moderate, high) does not align directly with SLIPTA’s official 0–5 star system, and the rationale for these categories is unclear.

**Recommendations:**

- Justify the use of these thresholds or consider reporting only continuous scores and standard SLIPTA star categories.
- Clearly state that the maximum possible SLIPTA score is 275 and relate observed scores to the official star levels.

**5. Data Collection Procedures and Quality Assurance**

The use of document review, observation, and structured questionnaires is appropriate. However, the manuscript lacks details on assessor training, standardization procedures, and quality assurance measures.

**Recommendations:**

- Report the number and qualifications of assessors.
- Describe any training or calibration exercises conducted.
- Indicate whether inter-rater reliability was assessed; if not, acknowledge this as a limitation.
- Clarify whether assessments were announced or unannounced and whether procedures were applied uniformly across facilities.

**6. Human Resource Norms and Staffing Analysis**

The comparison between expected and available laboratory personnel is informative, but the derivation of expected staffing levels is insufficiently explained.

**Recommendations:**

- Clearly state the source of staffing norms (e.g., Ministry of Health document and year).
- Describe how norms were applied across different facility types, including private and CHAG facilities.
- Clarify assumptions or adaptations and discuss how they may affect interpretation of staffing deficits and surpluses.

**7. Statistical Analysis and Reporting**

The use of non-parametric tests is appropriate; however, several statistical reporting issues need correction.

**Recommendations:**

- Report p-values appropriately (e.g., p < 0.001 instead of p = 0.00).
- Specify whether post-hoc pairwise comparisons were conducted following significant Kruskal–Wallis tests and report them if available.
- Where feasible, include measures of effect size or clearer interpretation of the magnitude of differences.
- Acknowledge the potential for type I error inflation due to multiple comparisons.

**8. Interpretation Beyond Collected Data**

The Discussion proposes explanations related to staff attitudes and resource allocation that were not directly measured in this study.

**Recommendations:**

- Clearly distinguish empirical findings from interpretations informed by the literature.
- Use cautious, non-causal language (e.g., “may reflect,” “could be attributable to”).
- Ensure appropriate citations accompany interpretive statements.

**9. Ethics and Consistency of Reporting**

There is inconsistency between the ethics information in the manuscript and the submission metadata, which indicates “N/A.”

**Recommendation:**

- Harmonize ethics reporting across the manuscript and submission system and briefly explain why ethical approval was required despite the absence of human participants or identifiable personal data.

**Minor Comments**

1. **Abstract:** Include key numerical results (median compliance score and range) and explicitly state that none of the laboratories met the minimum SLIPTA compliance threshold of 150 points, as this is a key finding.
2. **Abbreviations:** Define abbreviations at first use or include footnotes in tables (e.g., LMICs, UHC, SDGs, CHAG, NCCPA).
3. **Terminology:** Use consistent terminology throughout the manuscript (e.g., “quality laboratory standards,” “standard laboratory practice,” “quality management systems”).
4. **Tables:** Improve readability by standardizing formatting, indicating whether values are medians or means, and stating maximum possible scores for each domain.
5. **Discussion:** Correct the duplicated citation “(Mbah, 2014).”
6. **Language and Formatting:** Address minor grammatical and typographical issues and ensure references follow PLOS ONE style.

**Strengths of the Manuscript**

- Use of a validated international assessment framework (WHO SLIPTA).
- Census-based facility assessment, reducing selection bias.
- Detailed examination of quality system essentials and human resource distribution.
- High relevance for laboratory quality improvement and health policy in LMICs.
