# Supplementary material for: Assessment of compliance with quality laboratory standards in medical laboratories within the Kumasi Metropolis, Ghana
Source: PLoS One. 2026 Mar 20;21(3):e0335380. doi: 10.1371/journal.pone.0335380 (PMC13004333; doi:10.1371/journal.pone.0335380)
Supplement: Attachment — Submitted filename: Response to Reviewers.docx [file pone.0335380.s004.docx]

**PLOS ONE JOURNAL**

**USA, California**

**Dear Editor,**

**Response to Reviewers**

**Manuscript ID:** PONE-D-25-53279
**Title:** Assessment of compliance with quality laboratory standards in medical laboratories within the Kumasi Metropolis, Ghana
**Journal:** PLOS ONE

We would like to express our sincere gratitude to the Editor and Reviewers for their thorough, constructive, and insightful feedback. We appreciate their recognition of the importance of our study in improving laboratory systems in low- and middle-income countries. The manuscript has been meticulously revised to address all major and minor comments. Below, we provide a detailed, point-by-point response to each issue, outlining how we have incorporated their suggestions into the revised manuscript.

| **Reviewer** | **COMMENTS** | **RESPONSES** |
| --- | --- | --- |
| **Academic Editor** | | |
| Abstract level revisions | | |
| 1. | Corresponding author | The corresponding author has been indicated on the title page of the manuscript. |
| 2. | Facility Ownership and Classification | The Abstract has been revised to explicitly state the number of public and private standalone laboratories assessed.  **Location:** Abstract (lines 26-28) |
| 3. | Definition of Abbreviations | All abbreviations (e.g., SLIPTA, CHAG) are now defined at first mention.  **Location**: Abstract (lines 26-29) |
| 4. | SLIPTA Scoring Method | The Methods section now clearly explains how the scoring was done  **Location:** Methods (lines 30-34) |
| 5. | Reporting of p-values | All previously reported p-values of p = 0.00 have been corrected and are now reported as p < 0.001, in line with statistical reporting standards throughout the manuscript. |
| 6. | Alignment of Findings and Conclusions | The revised manuscript now explicitly states the expected score and its equivalent star rating; hence, the conclusion has been revised to align strictly with these findings.  **Location:** method (Lines 30-34); Conclusion (lines 42-43) |
| Introduction | | |
| 1. | Definition of abbreviations | All abbreviations are now defined at first mention in the Introduction. |
| 2. | Reference 10 and substantiation of Claims | Reference 10 has been corrected, and the statement is now supported by verifiable sources.  **Location:** Introduction (lines 92-34); References |
| 3. | Accreditation Institutions | The Introduction now explicitly lists relevant accreditation bodies.  **Location:** Introduction (lines 91-92) |
| 4. | Study Gap and Problem Statement | The Introduction has been strengthened to clearly articulate the knowledge and policy gap addressed by this study, specifically the lack of census-based data, including a detailed analysis of quality system essentials and staffing norms.  **Location:** Introduction (lines 85-96 and final paragraph) |
| Results | | |
| 1. | Expected MLS in Private Laboratories | We have clarified that the expected staffing levels for private laboratories were derived from adapted national health sector norms, and this explanation is now stated in the Methods and the table footnotes.  **Location:** Methods: Data collection (lines 164-171); Table 3 footnote |
| 2. | Statistical support for facility-level performance | A non-parametric pairwise comparison test has been performed to determine the best-performing facility.  **Location**: Result: Difference in laboratory compliance score by facility type (lines 241-255) |
| 3. | Column Clarity | Explanatory footnotes, including raw scores and average scores as applicable, have been added to tables.  **Location**: Table 6(foot note) |
| 4. | Reporting of p-values | All p-values in the Results section are now consistently reported as p < 0.001, where appropriate. |
| 5. | Discussion | The discussion has been revised to align with updated statistical analyses, ensuring that interpretations reflect the revised results without unsupported inferences. |
| 6. | Reference 10 Spelling and Accuracy | The bibliographic details for Reference 10 have been verified and corrected appropriately  Location: Introduction (Lines 85-87); Reference 19 and 20 |
| **Reviewers** | | |
| 1. | **SLIPTA Scoring, Categories, and Interpretation.**  “The manuscript reports overall and domain-specific compliance scores (e.g., a median score of 61) without clearly explaining how these relate to the maximum SLIPTA score of 275 or to the official SLIPTA star-rating system”. | The relationship between observed scores and the WHO SLIPTA system has been clearly established. A new table presenting the distribution of laboratories across SLIPTA star categories has been added. Non-standardized score ratings have been expunged from the manuscript.  **Location:**  Methods: Instrument (lines 138-142); Data analysis (Lines 176-185).  Results: Laboratory compliance scores by facility type (ines 217-218)  Table 5: Laboratory compliance scores by facility type |
| 2. | **Significance, Originality, and Contribution to the Literature.**  The study's added value relative to existing SLIPTA-based assessments is not sufficiently articulated. | The Introduction has been strengthened to clearly articulate the knowledge and policy gap addressed by this study, specifically the lack of census-based data, including a detailed analysis of quality system essentials and staffing norms.  **Location:** Introduction (lines 85-96 and final paragraph) |
| 3. | **Study Setting, Sampling Frame, and External Validity.**  The sampling frame and facility composition are insufficiently described, and the implications for generalisability are not discussed | We have clarified the sample frame and sampling procedures and included a discussion of their implications for external validity.  **Location**:  Method: Study population and sample (lines 124-128).  Strength and limitation (lines 335-342). |
| 4. | **Measurement Tool (SLIPTA)**  Non-standard compliance categories (low, moderate, high) do not align directly with SLIPTA’s official 0– star system | The relationship between observed scores and the WHO SLIPTA system has been clearly established. Non-standardized score ratings have been expunged from the manuscript.  **Location**:  Methods: Instrument (lines 138-142); Data analysis (Lines 176-185).  Results: Laboratory compliance scores by facility type (lines 217-218)  Table 5: Laboratory compliance scores by facility type |
| 5. | **Data Collection Procedures and Quality Assurance.**  Details on assessor training, standardization, and quality assurance are lacking | This has been clarified in the revised manuscript. The assessment was conducted by a licensed SLIPTA auditor.  **Location**:  Methods: Data collection (lines 150-152) |
| 6. | **Human Resource Norms and Staffing Analysis**  The derivation and application of expected staffing levels are insufficiently explained | We have revised the staffing analysis for clarity and described how norms were applied across government, CHAG, and private facilities, including the assumptions underlying those applications.  **Location**: Data collection (Lines 164-169); Table 3(footnote). |
| 7. | **Statistical Analysis and Reporting**  Statistical reporting requires correction and clarification. | We have revised the statistical reporting throughout the manuscript. Post hoc pairwise comparisons following significant Kruskal–Wallis tests are explicitly reported. The potential for type I error inflation due to multiple comparisons is acknowledged.  **Location**  Methods: Data analysis (183-185).  Results: Table 8  Limitations (lines 335-342) |
| 8. | **Interpretation Beyond Collected Data**  The Discussion proposes explanations related to staff attitudes and resource allocation that were not directly measured in this study. | We have revised the discussion to clearly distinguish empirical findings from literature-informed interpretations. |
| 9. | **Ethics and Consistency of Reporting**  There is an inconsistency between the ethics information in the manuscript and the submission metadata, which indicates “N/A.” | We have harmonised ethics reporting; ethics approval details are now consistent across the manuscript and the submission system. |

We are sincerely grateful to the Editor and Reviewers for the constructive feedback, which has greatly enhanced the clarity, rigour, and policy relevance of the manuscript. We hope the revised version now meets PLOS One’s publication criteria and look forward to your further consideration.

Sincerely,
Genevieve C. Aryeetey
